# Role of adhesion molecules and inflammation in Venezuelan equine encephalitis virus infected mouse brain

**DOI:** 10.1186/1743-422X-8-197

**Published:** 2011-04-29

**Authors:** Anuj Sharma, Manish Bhomia, Shelley P Honnold, Radha K Maheshwari

**Affiliations:** 1 Dept of Pathology, Uniformed Services University of the Health Sciences, Bethesda, MD, USA; 2 Biological Sciences Group, Birla Institute of Technology and Science, Pilani, India

## Abstract

**Background:**

Neuroinvasion of Venezuelan equine encephalitis virus (VEEV) and subsequent initiation of inflammation in the brain plays a crucial role in the outcome of VEEV infection in mice. Adhesion molecules expressed on microvascular endothelial cells in the brain have been implicated in the modulation of the blood brain barrier (BBB) and inflammation in brain but their role in VEEV pathogenesis is not very well understood. In this study, we evaluated the expression of extracellular matrix and adhesion molecules genes in the brain of VEEV infected mice.

**Findings:**

Several cell to cell adhesion molecules and extracellular matrix protein genes such as ICAM-1, VCAM-1, CD44, Cadherins, integrins, MMPs and Timp1 were differentially regulated post-VEEV infection. ICAM-1 knock-out (IKO) mice infected with VEEV had markedly reduced inflammation in the brain and demonstrated a delay in the onset of clinical symptoms of disease. A differential regulation of inflammatory genes was observed in the IKO mice brain compared to their WT counterparts.

**Conclusions:**

These results improve our present understanding of VEEV induced inflammation in mouse brain.

## Findings

**Figure 1:**
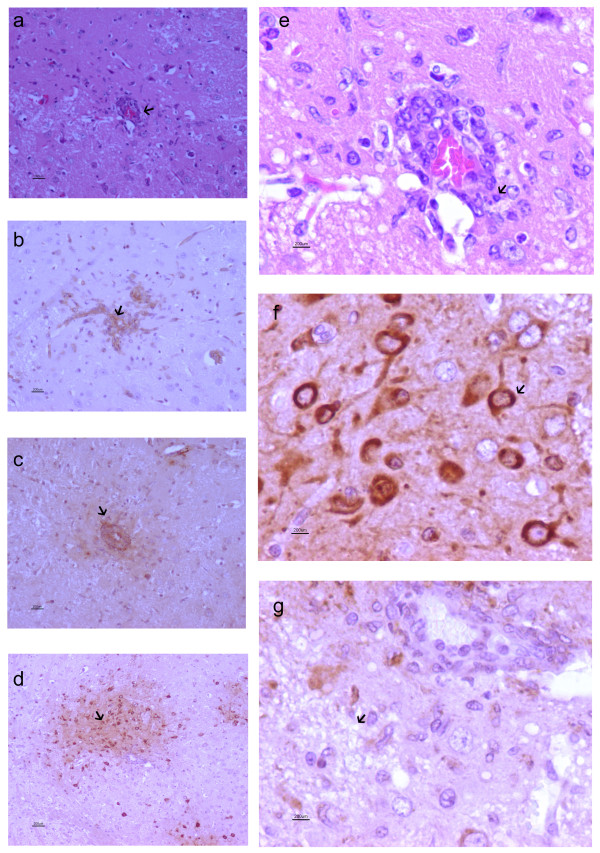
**ICAM-1 expression corresponds with perivascular cuffing, fibrinogen leakage, and the presence of VEEV in the brains of VEEV infected mice**. (a) H&E staining of the brain of VEEV infected CD-1 mice exhibited prominent perivascular cuffs throughout the brain. (b) Endothelial cells are hypertrophied and exhibit positive immunoreactivity for ICAM-1. (c) Immunohistochemistry for fibrinogen demonstrated localized fibrinogen leakage around hypertrophied endothelial cells, indicating disruption of the BBB. (d) Extensive VEEV specific staining revealed numerous cells infected with VEEV around the affected vessels. (e) Perivascular cuffs were composed of moderate numbers of mononuclear cells, primarily lymphocytes and fewer monocytes which had transmigrated the endothelium and multifocally extended into the neuropil. (f) Neurons were primarily infected with VEEV; however, VEEV antigen was also localized in glia and fewer lymphocytes. (g) Vacuolation of the neuropil was observed around the affected vessels.

**Figure 2:**
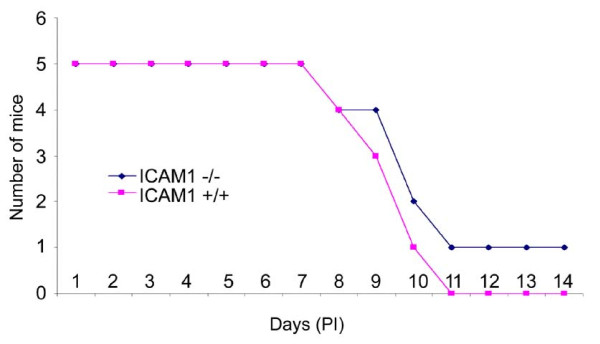
**Survival study in IKO mice**. Representative of two separate survival studies done in IKO mice upon VEEV infection is shown here. Mice were observed twice a day for fourteen days pi for signs of clinical disease. There was a 20% reduction in mortality in IKO mice over their WT controls in both the studies.

**Figure 3:**
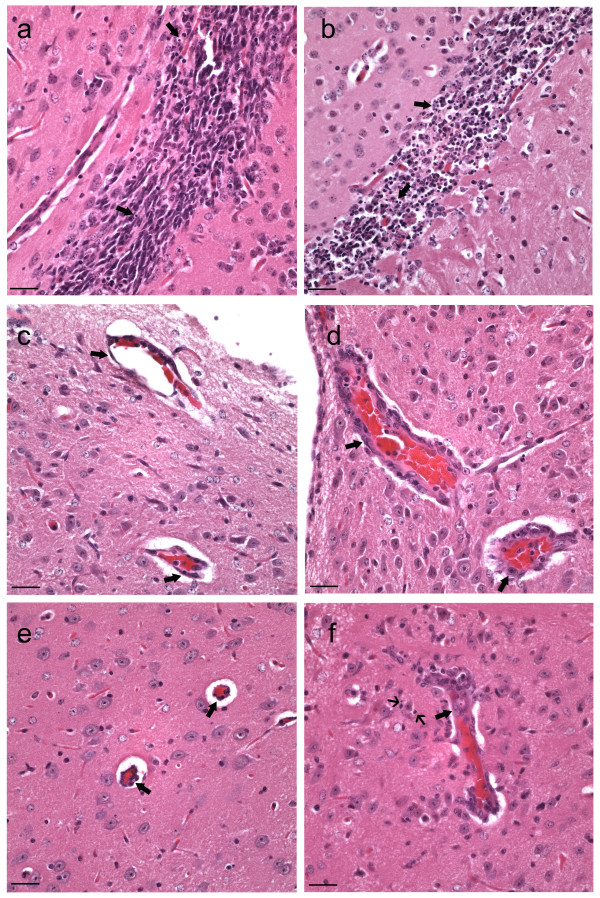
**Inflammation in the brain of VEEV infected IKO and WT mice**. (a) IKO 96 hr pi cerebrum, subventricular zone, H&E, 400X: There were low numbers of necrotic neurons, characterized by small amounts of karyorrhexis (arrows), when compared to the same region of the WT mice (b). (b) WT 96n hr pi, cerebrum, subventricular zone, H&E, 400X: There were high numbers of necrotic neurons, characterized by large amounts of karyorrhexis (arrows), when compared to the same region of IKO mice (a). (c) IKO 96 hr pi, brainstem, H&E, 400X: There was little to no endothelial swelling and no perivascular cuffing (arrows) compared to the WT mice (d). (d) WT 96 hr pi, brainstem, H&E, 400X: There was moderate endothelial swelling and mild perivascular cuffing (thick arrows) and rare necrotic cells (thin arrow) compared to the IKO mice (c). (e) IKO 96 hr pi, brain, thalamus, H&E, 400X: There was little to no endothelial swelling and no perivascular cuffing (arrows) compared to the WT mice (f). (f) WT 96 hr pi, brain, thalamus, H&E, 400X: There was moderate endothelial swelling and mild perivascular cuffing (thick arrow) and rare necrotic cells (thin arrows) compared to the IKO mice (e).

**Figure 4:**
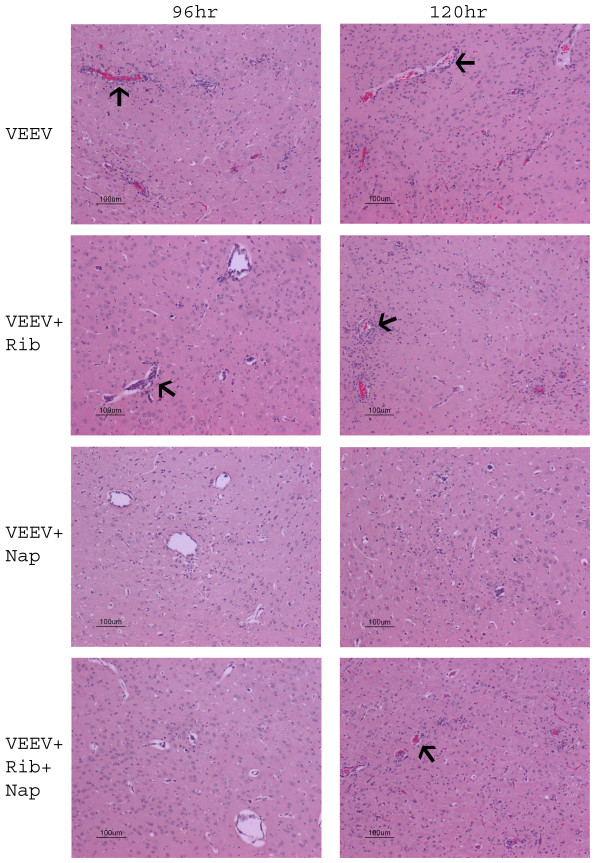
**Inflammation was reduced in VEEV infected mice treated with naproxen or naproxen plus ribavirin**. Animals were treated with naproxen (40 mg/kg/once a day) only or naproxen plus ribavirin (80 mg/kg/once a day) at the time of infection (1000 pfu of VEEV inoculated in left rear foot pad). Animals were monitored twice a day and clinical symptoms of disease were monitored for two weeks.

**Figure 5:**
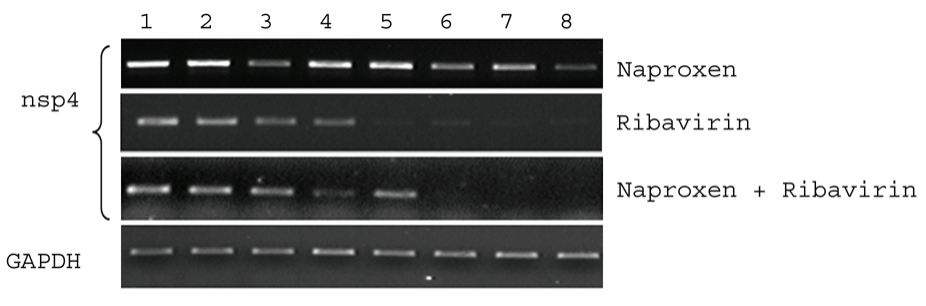
**Viral load in the brain of mice treated with naproxen and naproxen plus rivavirin**. Animals were treated with naproxen and naproxen plus ribavirin as described in the text. Mice were sacrificed at each given time points (n = 5 each group) and brain tissues were evaluated for viral load by RT-PCR. Given is a representative picture of one of the set of the mice. (1) drug treated 120 hr pi; (2) untreated 120 hr pi; (3) drug treated 96 hr pi; (4) untreated 96 hr pi; (5) drug treated 72 hr pi; (6) untreated 72 hr pi; (7) drug treated 48 hr pi; and (8) untreated 48 hr pi.

Neurovirulent Venezuelan equine encephalitis virus (VEEV) is a member of the genus *Alphavirus*, in the family *Togaviridae*. VEEV causes lethal encephalitis in equines and occasionally infect humans [1,2]. In our earlier studies, we have reported upregulation of several integrins (Itg) and integrin binding molecule genes such as nischarin (Nisch) and integrin alpha V (ItgaV) as well as genes that are implicated in the alteration of the blood brain barrier (BBB) such as MCP-1 [3] in the brains of VEEV infected mice [4,5]. Other studies have also shown that adhesion molecules expressed on the surface of microvascular endothelial cells of the BBB play an important role in viral encephalitis [6,7]. VEEV replicon particles have also been recently reported to disrupt BBB [8]. Though, VEEV has been known to enter the central nervous system (CNS) through the olfactory tract, these studies indicate that adhesion molecules expressed on the BBB may also play role in VEEV pathogenesis [2]. Therefore, in this study, we evaluated the expression of extracellular matrix (ECM) and adhesion molecules in VEEV infected CD-1 mice brain. Several ECM and adhesion molecules genes, such as integrins (ItgαX, Itg2, 3, and 7), cadherin (Cdh) 1 and 2, intracellular adhesion molecule-1 (ICAM-1), and vascular cell adhesion molecule (VCAM-1) were found to be upregulated in the brains of VEEV infected CD-1 mice (Table 1). Immunohistochemistry analysis showed ICAM-1 expression and its co-localization with inflammation, fibrinogen leakage and VEEV antigen in and around the brain microvessels (Figure 1). Pathway analysis of the modulated ECM and adhesion molecules genes using DAVID software [9,10] indicated their involvement in leukocyte migration at BBB (Additional file 1 Figure S1). Of these several genes, ICAM-1 has been implicated in the pathogenesis of various other neurotropic viruses such as West Nile virus, Semliki Forest virus, Theiler's murine encephalomyelitis virus and lymphocyte choriomeningitis virus [11-14]. To test if ICAM-1 plays any role in VEEV pathogenesis, ICAM-1 knockout (IKO) mice (B6.129S4-Icam1tm1Jcgr/J, stock No. 002867, Jackson Laboratories, Bar Harbor, Maine, USA) were infected with 1000 pfu of VEEV in two separate studies. In both these studies, VEEV infected wild type (WT; C57BL/6J, stock No. 000664, Jackson Laboratories, Bar Harbor, Maine, USA) mice became sick earlier than the ICAM-1 knock-out (IKO) mice. IKO mice showed delayed appearance of the clinical symptoms such as shivering, excitability, and hind limb paralysis with a total loss of mobility. General health and appearance of the WT mice evaluated as ruffled fur, hunched back posture and lethargy in the early stages of the disease was severe than similarly infected IKO mice. IKO mice were more responsive to touch, less lethargic and were eating better than the WT mice. Though a 20% reduction in mortality was observed in both the studies (Figure 2), there was no difference in the mean survival time of the IKO mice that succumbed to VEEV infection as compared to the WT mice. Histopathological analysis of brain showed less severe single cell necrosis and perivascular cuffing in the IKO mice brain than the WT mice at 96 hr post infection (pi) (Figure 3). However, no significant difference in VEEV antigen was noticed (data not shown) in the brains of IKO and WT mice. The inflammatory cytokine specific focused microarray performed on the brain RNA samples of VEEV infected WT and IKO mice at 96 hr pi (Table 2) showed inflammatory gene expression differences in the brain of IKO mice. Basal level differences in the cytokine expression of uninfected IKO and WT brain are given in additional file 2 Table S1. Microarray analysis (Table 2) showed a complex inflammatory response to VEEV infection in the brain of IKO mice which can be classified based on pro- and anti-inflammatory response and may explain some of the differences seen early in the infection of IKO and WT mice. Several pro-inflammatory cytokines/ligands such as Ccl24, Cxcl11 and Cxcl13 were down regulated in IKO mice brain as compared to WT mice. Ccr2, a chemokine receptor that is involved in the migration of peripheral blood mononuclear cells into the brain [15] was also down regulated in the brains of VEEV infected IKO mice. Anti-inflammatory genes IL10, Ccl22 and IL22 were also down regulated in the brains of VEEV infected IKO mice. These cytokines and chemokines have been implicated in the regulation and suppression of inflammation in the tissues [16-19]. Other pro-inflammatory genes such as Ccr1 and Xcl1 that have been implicated in the recruitment of mononuclear phagocytes and T cells respectively into the brain [20-23] were either upregulated or induced in the VEEV infected IKO mice brain. Modulation of these anti-inflammatory and pro-inflammatory genes may contribute to the final outcome of inflammation observed in the brain of VEEV infected IKO mice. Gene expression evaluation at early and later time points than 96 hr pi may shed more light on the inflammatory gene expression kinetics in the brain of IKO mice. To further evaluate if inflammation is crucial, animals were treated with a known anti-inflammatory drug naproxen (40 mg/kg, once a day). Similar to the VEEV infected IKO mice, naproxen treated VEEV infected CD-1 mice (n = 10) early in the infection (0-5 days) showed lesser degree of ruffled fur, lethargy, hunched back, and delayed appearance of shivering and paralysis as compared to the untreated mice (n = 10), thereafter, these mice quickly became sick and there was no difference in the mean survival time of naproxen treated and untreated VEEV infected mice. A marked decrease but not total ablation in the vascular inflammation was observed in the naproxen treated VEEV-infected mice at 96 and 120 hr pi (n = 3 each group) (Figure 4). Viral load in the brain was evaluated by RT-PCR at 48, 72, 96 and 120 hr pi (n = 5 each group). There was no significant difference in the viral load in the brain of the naproxen treated and untreated VEEV infected mice groups. However, when taken as individual sets of mice, more viral load was observed in the brain of some of the naproxen treated mice as compared to the untreated ones (Figure 5). Treatment with ribavirin (80 mg/kg, once a day), a known anti-viral drug, alone or in combination with naproxen also did not affect the mortality or the viral load in VEEV infected mice. These results show a complex role of inflammation in VEEV pathology, initial better health of naproxen treated mice may be due to reduced inflammation in the brain. However, increase in brain viral load in naproxen treated animals may be due to unchecked viral replication due to reduced inflammation.

**Table 1:** Mouse extracellular matrix and adhesion molecules gene(s) differentially expressed by two fold in VEEV infected CD 1 mouse brain.

*Position on Array*	*Ref Sequence Number*	*Functional Grouping (Gene)*	*Fold expression over uninfected controls*			
			
			*48 hr pi*	*72 hr pi*	*96 hr pi*	*120 hr pi*
***Cell Adhesion molecules: Transmembrane/cell-cell adhesion/cell-matrix adhesion/other molecules***						

9	NM_009851	CD44 antigen (Cd44)	(A)	5.24 (PI) ± 4.24 (0.37)	16.35 (PI) ± 10.09 (0.20)	34.83 (PI) ± 3.23 (0.0004)

10	NM_009864	Cadherin 1 (Cdh1)	3.97 ± 0.32 (0.0007)	4.98 ± 3.37 (0.30)	6.52 ± 5.23 (0.35)	16.79 ± 1.57 (0.0005)

14	NM_009868	Cadherin 5 (Cdh5)	(A)	1.17 (PI) ± 0.17 (0.37)	1.92 (PI) ± 0.59(0.19)	9.57 (PI) ± 0.97 (0.0009)

29	NM_016919	Procollagen, type V, alpha 3 (Col5a3)	0.81 ± 0.15 (0.51)	1.09 ± 0.19 (0.78)	1.60 ± 0.67 (0.16)	2.07 ± 0.31 (0.03)

32	NM_007739	Procollagen, type VIII, alpha 1 (Col8a1)	1.41 ± 0.49 (0.53)	1.77 ± 1.31(0.27)	1.18 ± 0.39 (0.70)	0.80 ± 0.61 (0.65)

34	XM_488510/ NM_001081249.1	Mus musculus chondroitin sulfate proteoglycan 2(Cspg2)/Versican (Vcan)	1.14 ± 0.40 (0.74)	2.36 ± 0.80 (0.28)	3.34 ± 1.13 (0.23)	4.39 ± 1.83 (0.07)

35	NM_010217	Connective tissue growth factor (Ctgf)	0.88 ± 0.13 (0.38)	0.91 ± 0.13 (0.52)	0.83 ± 0.05 (0.24)	0.45 ± 0.10 (0.01)

38	NM_009848	Ectonucleoside triphosphate diphosphohydrolase 1(Entpd1)	(A)	(A)	(A)	4.37 (PI) ± 1.41 (0.03)

41	NM_013500	Hyaluronan and proteoglycan link protein 1 (Hapln1)	0.67 (PI) ± 0.29	1.15 (PI) ± 0.13 (0.73)	(A)	(A)

43	NM_010493	Intercellular adhesion molecule (Icam1)	2.82 (PI) ± 1.32 (0.24)	8.4 (PI) ± 3.00 (0.07)	13.51 (PI) ± 6.24 (0.12)	36.76 (PI) ± 4.81 (0.001)

56	NM_021334	Integrin alpha × (Itgax)	(A)	(A)	4.49 ± 1.12 (0.01)	3.55 (PI) ± 0.89 (0.02)

57	NM_010578	Integrin beta 1 (fibronectin receptor beta) (Itgb1)	1.06 ± 0.48 (0.83)	1.61 ± 0.35 (0.27)	2.41 ± 0.56 (0.02)	2.67 ± 0.39 (0.01)

58	NM_008404	Integrin beta 2 (Itgb2)	(A)	(A)	(A)	4.79 (PI) ± 0.27 (0.0.0001)

59	NM_016780	Integrin beta 3 (Itgb3)	1.36 (PI) ± 0.25 (0.59)	2.32 (PI) ± 0.30 (0.10)	3.37 (PI) ± 1.32 (0.03)	3.06 (PI) ± 0.55 (0.01)

63	NM_013566	Integrin beta 7 (Itgb7)	0.83 ± 0.13 (0.50)	(A)	3.40 ± 1.98 (0.27)	7.84 (PI) ± 3.04 (0.04)

96	NM_011346	Selectin, lymphocyte (Sell)	2.67 (PI) ± 0.89 (0.33)	10.06 (PI) ± 5.41 (0.08)	20.37(PI) ± 11.02 (0.07)	21.45 (PI) ± 5.60 (0.001)

97	NM_011347	Selectin, platelet (Selp)	12.66 (PI) ± 8.84 (0.26)	32.57 (PI) ± 2.66 (0.0002)	43.14 (PI) ± 5.70 (0.001)	37.60 (PI) ± 4.37 (0.001)

105	NM_011581	Thrombospondin 2 (Thbs2)	1.17 ± 0.34 (0.58)	1.60 ± 0.82 (0.44)	2.87 ± 1.58 (0.40)	4.02 ± 3.50 (0.39)

107	NM_011582	Thrombospondin 4 (Thbs4)	11.99 ± 3.25 (0.03)	2.70 ± 1.19 (0.22)	1.30 ± 0.30 (0.37)	(A)

***Extracellular Matrix Proteins: Basement membrane constituents/ECM proteases and their inhibitors/others***						

5	NM_013906	A disintegrin-like and metallopeptidase (reprolysin type) with thrombospondin type 1 motif, 8 (Adamts8)	(A)	2.44 (PI) ± 0.34 (0.01)	(A)	2.78 (PI) ± 0.41 (0.01)

16	NM_007729	Procollagen, type XI, alpha 1 (Col11a1)	(A)	2.60 ± 1.61(0.37)	(A)	(A)

77	NM_008608	Matrix metallopeptidase 14 (membrane-inserted) (Mmp14)	0.51 ± 0.09 (0.05)	1.07 ± 0.12 (0.76)	1.32 ± 0.28 (0.11)	1.24 ± 0.20 (0.17)

78	NM_008609	Matrix metallopeptidase 15 (Mmp15)	(A)	(A)	2.48 ± 1.48 (0.37)	(A)

79	NM_019724	Matrix metallopeptidase 16 (Mmp16)	0.49 ± 0.16 (0.09)	1.62 ± 0.07 (0.22)	0.68 ± 0.26 (.035)	1.18 ± 0.32 (0.65)

87	NM_010809	Matrix metallopeptidase 3 (Mmp3)	4.60 (PI) ± 3.34 (0.34)	29.25 (PI) ± 8.41 (0.03)	48.06 (PI) ± 2.98 (0.00009)	26.60 (PI) ± 5.76 (0.01)

89	NM_008611	Matrix metallopeptidase 8 (Mmp8)	1.21 ± 0.29 (0.46)	(A)	6.15 ± 4.73 (0.38)	22.59 (PI) ± 6.94 (0.05)

101	NM_009263	Secreted phosphoprotein 1 (Spp1)	1.00 ± 0.03 (0.99)	0.67 ± 0.18 (0.12)	0.63 ± 0.17 (0.12)	0.50 ± 0.06 (0.01)

103	NM_009369	Transforming growth factor, beta induced (Tgfbi)	0.87 ± 0.10 (0.40)	0.55 ± 0.19 (0.15)	0.06 ± 0.04 (0.001)	0.08 ± 0.06 (0.001)

108	NM_011593	Tissue inhibitor of metalloproteinase 1 (Timp1)	(A)	4.88 (PI) ± 2.38 (0.18)	17.64 (PI) ± 5.88 (0.05)	13.79 (PI) ± 1.36 (0.0007)

112	NM_011607	Tenascin C (Tnc)	(A)	(A)	0.49 ± 0.25 (0.37)	(A)

Genes that are differentially expressed by ≥2.0 fold are represented. Values are represented at mean fold difference ± SEM (P-value). (A) = absent; PI = present in infection and; PC = present in saline control.

**Table 2:** Differential gene expression in brains of VEEV infected ICAM-1 WT mice (n = 3) as compared to IKO mice (n = 3) at 96 hr pi.

*Position*	*RefSeq Number*	*Symbol*	*Description*	*IKO Mean±SEM*	WT *Mean±SEM*	*Fold Change* *(IKO/WT)*
7	NM_011332	Ccl17	Chemokine (C-C motif) ligand 17 (Ccl17)	73.57	(A)	P-IKO

13	NM_019577	Ccl24	Chemokine (C-C motif) ligand 24 (Ccl24)	(A)	273.69 POR	P-WT

22	NM_009912	Ccr1	Chemokine (C-C motif) receptor 1 (Ccr1)	132.24 ± (40.75)	28.80 POR	4.59

35	NM_019494	Cxcl11	Chemokine (C-X-C motif) ligand 11 (Cxcl11)	435.04 ± (167.36)	690.98 ± (322.64)	0.63

37	NM_018866	Cxcl13	Chemokine (C-X-C motif) ligand 13 (Cxcl13)	163.29 POR	245.09 ± (156.61) PTwR	0.67

49	NM_008337	Ifng	Interferon gamma (Ifng)	211.35 ± (21.67) PTwR	122.81 ± (12.68) PTwR	1.72

51	NM_010548	Il10	Interleukin 10 (Il10)	(A)	57.40 ± (43.51) PTwR	P-WT

68	NM_008362	Il1r1	Interleukin 1 receptor, type I (Il1r1)	119.93 POR	(A)	P-IKO

69	NM_010555	Il1r2	Interleukin 1 receptor, type II (Il1r2)	287.27 POR	172.19 ± (94.76) PTwR	1.67

73	NM_016971	Il22	Interleukin 22 (Il22)	(A)	186.84 POR	P-WT

74	NM_008368	Il2rb	Interleukin 2 receptor, beta chain (Il2rb)	151.12 POR	31.83 POR	4.75

93	NM_011101	Prkca	Protein kinase C, alpha (Prkca)	60.8 POR	(A)	P-IKO

94	NM_009007	Rac1	RAS-related C3 botulinum substrate 1 (Rac1)	195.79 ± (6.81) PTwR	105.57 ± (81.61) PTwR	1.85

95	NM_007926	Scye1	Small inducible cytokine subfamily E, member 1 (Scye1)	385.53 ± (137.18)	243.61 ± (159.47)	1.58

96	NM_009263	Spp1	Secreted phosphoprotein 1 (Spp1)	693.01 ± (74.26)	389.60 ± (127.58)	1.78

104	NM_133211	Tlr7	Toll-like receptor 7 (Tlr7)	176.40 ± (87.47) PTwR	35.74 ± (22.32) PTwR	4.94

107	NM_013693	Tnf	Tumor necrosis factor (Tnf)	74.90 ± (8.85) PTwR	193.78 POR	0.39

112	NM_008510	Xcl1	Chemokine (C motif) ligand 1 (Xcl1)	76.09 ± (42.81) PTwR	(A)	P-IKO

Average expression values of gene are given. Where gene expression was detected in only one biological sample and not the replicates, the gene expression value is followed by POR (present in one replicate), similarly where the gene expression was detected in two biological replicates the gene expression value is followed by PTwR (present in two replicates). The fold expression values are derived by dividing average expression (or expression value from one or two replicates) of VEEV infected IKO samples with average expression (or expression value from one replicate) of VEEV infected ICAM *WT *samples and vice versa. PI = Expressed only in VEEV infected samples, PC = Expressed in uninfected saline control samples only, (A) = absent. Values are expressed as ± SEM.

To our knowledge, this is the first study to evaluate the expression of ECM and adhesion molecules in brain during VEEV infection and to evaluate the inflammatory response in IKO mice during VEEV infection. The results show that adhesion molecules such as ICAM-1 may play an important role in VEEV disease pathology. These findings improve our present understanding of VEEV induced inflammation in the mouse brain and its implication in VEEV disease.

## Competing interests

The authors declare that they have no competing interests.

## Authors' contributions

AS participated in the study design, carried out the animal experiments, performed microarray, PCR experiments and performed statistical analysis. MB carried out tissue processing, PCR experiments and performed statistical analysis. SPH analyzed histology slides. RKM conceived of the study, participated in the study design and coordinated and helped to draft the manuscript. All authors read and approved the final manuscript.

## Supplementary Material

Additional file 1 **Figure S1: Functional pathway analysis of adhesion molecules expressed in the brain of VEEV infected mice**. All the ECM protein and adhesion molecule genes that were differentially modulated in the brains of VEEV infected mice were subjected to pathway analysis by DAVID software functional annotation tool. The genes that are circled in red were differentially regulated in this study. The diagram shows their location and involvement in leukocyte transendothelial migration at tight junctions. Click here for file

Additional file 2 **Table S1: Differential gene expression in the brain of uninfected, saline injected WT mice (n = 2) as compared to uninfected, saline injected IKO mice (n = 2)**. Average expression values of each gene are given. Where gene expression was detected in only one biological sample and not the replicates, the gene expression value is followed by POR (present in one replicate). The fold expression values are derived by dividing average (or expression value from one replicate) expression of IKO controls with average (or expression value from one replicate) expression of ICAM-1 *WT *(WT) samples. P-IKO = Expressed only in IKO samples only, P-WT = present in WT samples only, (A) = absent. Values are expressed as ± SEM. Click here for file
