# Supplementary figures and images for: Role of adhesion molecules and inflammation in Venezuelan equine encephalitis virus infected mouse brain
Source: Virol J. 2011 Apr 29;8:197. doi: 10.1186/1743-422X-8-197 (PMC3113303; doi:10.1186/1743-422X-8-197)

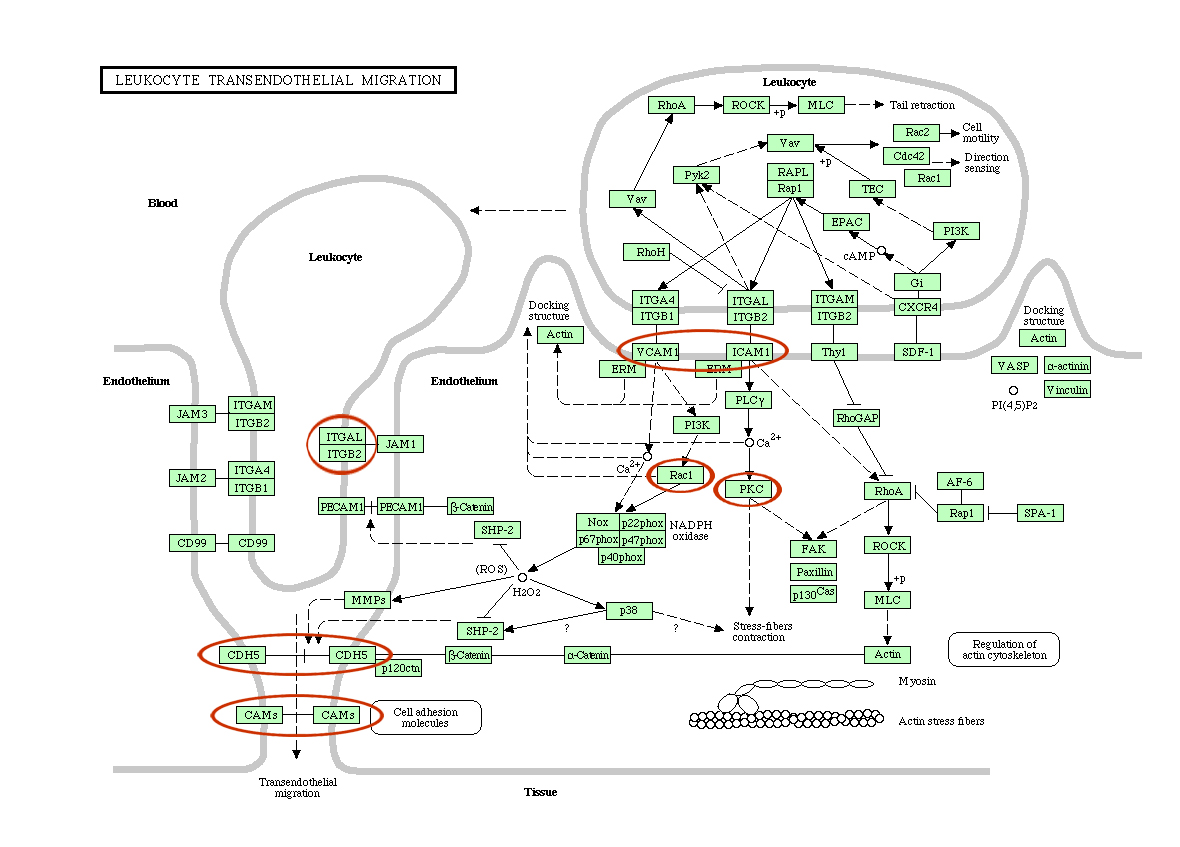

Supplement: Additional file 1 — Figure S1: Functional pathway analysis of adhesion molecules expressed in the brain of VEEV infected mice. All the ECM protein and adhesion molecule genes that were differentially modulated in the brains of VEEV infected mice were subjected to pathway analysis by DAVID software functional annotation tool. The genes that are circled in red were differentially regulated in this study. The diagram shows their location and involvement in leukocyte transendothelial migration at tight junctions. [file 1743-422X-8-197-S1.JPEG]
