# Supplementary material for: Role of adhesion molecules and inflammation in Venezuelan equine encephalitis virus infected mouse brain
Source: Virol J. 2011 Apr 29;8:197. doi: 10.1186/1743-422X-8-197 (PMC3113303; doi:10.1186/1743-422X-8-197)
Supplement: Additional file 2 — Table S1: Differential gene expression in the brain of uninfected, saline injected WT mice (n = 2) as compared to uninfected, saline injected IKO mice (n = 2). Average expression values of each gene are given. Where gene expression was detected in only one biological sample and not the replicates, the gene expression value is followed by POR (present in one replicate). The fold expression values are derived by dividing average (or expression value from one replicate) expression of IKO controls with average (or expression value from one replicate) expression of ICAM-1 WT (WT) samples. P-IKO = Expressed only in IKO samples only, P-WT = present in WT samples only, (A) = absent. Values are expressed as ± SEM. [file 1743-422X-8-197-S2.DOC]

**Additional file 2, Table S1:** Differential gene expression in the brain of uninfected, saline injected WT mice (n=2) as compared to uninfected, saline injected IKO mice (n=2).

| ***Position*** | ***RefSeq Number*** | ***Description*** | ***WT Control Mean*± *(SEM)*** | ***IKO Control Mean± SEM*** | ***Fold Difference*** |
| --- | --- | --- | --- | --- | --- |
| 8 | NM_011888 | Chemokine (C-C motif) ligand 19 (Ccl19) | (A) | 120.72 ± (3.88) | P-IKO |
| 12 | NM_009137 | Chemokine (C-C motif) ligand 22 (Ccl22) | 507.26 ±(462.92) | 100.25 ± (32.00) | 0.20 |
| 23 | NM_009915 | Chemokine (C-C motif) receptor 2 (Ccr2) | 308.15 ±(197.23) | 483.70 ± (443.73) | 1.57 |
| 28 | NM_007719 | Chemokine (C-C motif) receptor 7 (Ccr7) | (A) | 86.28 POR | P-IKO |
| 31 | NM_009142 | Chemokine (C-X3-C motif) ligand 1 (Cx3cl1) | 140.56 ±(67.23) | 227.63 ± (168.36) | 1.62 |
| 32 | NM_009987 | Chemokine (C-X3-C) receptor 1 (Cx3cr1) | 286.58 ±(96.77) | 631.26 ± (50.97) | 2.20 |
| 51 | NM_010548 | Interleukin 10 (Il10) | 82.97 POR | 485.66 POR | 5.85 |
| 58 | NM_008355 | Interleukin 13 (Il13) | (A) | 337.20 POR | P-IKO |
| 67 | NM_008361 | Interleukin 1 beta (Il1b) | 118.42 POR | (A) | P-WT |
| 73 | NM_016971 | Interleukin 22(Il22) | (A) | 545.22 POR | P-IKO |
| 76 | NM_010556 | Interleukin 3(Il3) | (A) | 123.08 POR | P-IKO |
| 94 | NM_009007 | RAS-related C3 botulinum substrate 1 (Rac1) | 139.62 ±(14.32) | 214.68 ± (64.82) | 1.54 |
| 100 | NM_126166 | Toll-like receptor 3 (Tlr3) | 69.44 ±(52.66) | 48.32 ± POR | 0.70 |
| 108 | NM_011609 | Tumor necrosis factor receptor superfamily, member 1a (Tnfrsf1a) | (A) | 107.71 POR | P-IKO |

Average expression values of each gene are given. Where gene expression was detected in only one biological sample and not the replicates, the gene expression value is followed by POR (present in one replicate). The fold expression values are derived by dividing average (or expression value from one replicate) expression of ICAM-1 knock out (IKO) controls with average (or expression value from one replicate) expression of ICAM-1 *WT* (WT) samples. P-IKO= Expressed only in IKO samples only, P-WT= present in WT samples only, (A) = absent. Values are expressed as ± SEM.
